# Evaluation of Medicaid Expansion Under the Affordable Care Act and Contraceptive Care in US Community Health Centers

**DOI:** 10.1001/jamanetworkopen.2020.6874

**Published:** 2020-06-04

**Authors:** Blair G. Darney, R. Lorie Jacob, Megan Hoopes, Maria I. Rodriguez, Brigit Hatch, Miguel Marino, Anna Templeton, Jee Oakley, Erika K. Cottrell

**Affiliations:** 1 Oregon Health & Science University, Portland, Oregon; 2 OHSU-PSU School of Public Health, Portland, Oregon; 3 National Institute of Public Health, Center for Population Health, Cuernavaca, Morelos, Mexico; 4 OCHIN Inc, Portland, Oregon

## Abstract

**Importance:**

Use of effective contraception decreases unintended pregnancy. It is not known whether Medicaid expansion under the Affordable Care Act increased use of contraception for women who are underserved in the US health care safety net.

**Objective:**

To evaluate the association of Medicaid expansion under the Affordable Care Act with changes in use of contraception among patients at risk of pregnancy at US community health centers, with the hypothesis that Medicaid expansion would be associated with increases in use of the most effective contraceptive methods (long-acting reversible contraception).

**Design, Setting, and Participants:**

This was a participant-level retrospective cross-sectional study comparing receipt of contraception before (2013) vs immediately after (2014) and a longer time after (2016) Medicaid expansion. Electronic health record data from a clinical research network of community health centers across 24 states were included. The sample included all female patients ages 15 to 44 years at risk for pregnancy, with an ambulatory care visit at a participating community health center during the study period (315 clinics in expansion states and 165 clinics in nonexpansion states).

**Exposures:**

Medicaid expansion status (by state).

**Main Outcomes and Measures:**

Two National Quality Forum–endorsed contraception quality metrics, calculated annually: the proportion of women at risk of pregnancy who received (1) either a moderately effective or most effective method (hormonal and long-acting reversible contraception) methods and (2) the most effective method (long-acting reversible contraception).

**Results:**

The sample included 310 132 women from expansion states and 235 408 women from nonexpansion states. The absolute adjusted increase in use of long-acting reversible contraceptive methods was 0.58 (95% CI, 0.13-1.05) percentage points greater among women in expansion states compared with nonexpansion states in 2014 and 1.19 (95% CI, 0.41-1.96) percentage points larger in 2016. Among adolescents, the association was larger, particularly in the longer term (2014 vs 2013: absolute difference-in-difference, 0.80 [95% CI, 0.30-1.30] percentage points; 2016 vs 2013: absolute difference, 1.79 [95% CI, 0.88-2.70] percentage points). Women from expansion states who received care at a Title X clinic had the highest percentage of women receiving most effective contraceptive methods compared with non–Title X clinics and nonexpansion states.

**Conclusions and Relevance:**

In this study, Medicaid expansion was associated with an increase in use of long-acting reversible contraceptive methods among women at risk of pregnancy seeking care in the US safety net system, and gains were greatest among adolescents.

## Key Points

**Question:**

Was Medicaid expansion under the Affordable Care Act associated with improvements in contraceptive use quality metrics in the US health care safety net?

**Findings:**

In this cross-sectional study including more than 500 000 women, Medicare expansion was associated with an increase in use of the most effective contraceptive methods (long-acting reversible contraception) by 1.2 percentage points among women at risk of pregnancy in states that expanded Medicaid compared with nonexpansion states, controlling for other payers for contraceptive care (such as Title X and 1115 waiver programs).

**Meaning:**

Affordable Care Act–sponsored Medicaid expansion was independently associated with a small but meaningful increase in access to the most effective methods of contraception.

## Teaser

This cross-sectional study evaluates the association of Medicaid expansion under the Affordable Care Act with changes in use of contraception among patients at risk of pregnancy receiving care at US community health centers.

## Introduction

Unintended pregnancy is a key health indicator with multigenerational negative social, economic, and health consequences.^1^ The proportion of unintended pregnancies overall is declining, but disparities by race/ethnicity, income, and insurance status persist.^2,3,4^ Women with low incomes and a lack of insurance have double the burden of unintended pregnancy compared with women with incomes of 200% or more than the federal poverty level (FPL).^5^ Use of effective contraception is an essential intervention to reduce unintended pregnancy. Community health centers (CHCs), which include federally qualified health centers, rural health centers, and county health departments, receive federal funds to provide care regardless of insurance status or ability to pay and are key sources of care for women of reproductive age.^6^ In 2015, more than 6 million women in the US received publicly funded contraceptive services in CHCs or other safety net settings.^7^

The Affordable Care Act (ACA) increased the number of adults receiving health insurance coverage, especially in Medicaid expansion states,^8,9,10^ and insurance coverage for preventive services, including contraception, without any out-of-pocket costs to patients.^11^ Recent studies suggest that the number of women receiving contraceptive services in primary care settings increased in Medicaid expansion states after ACA implementation,^12,13,14^ and more women in safety net clinics used insurance to pay for contraceptive services after Medicaid expansion.^15^ Research to date on publicly funded contraceptive services has focused on 1 of 3 mechanisms (Medicaid, single payers [eg, state family-planning programs],^16^ or federal programs [Title X]^17,18,19^) or has included only clinics with a reproductive health focus or those known to provide publicly funded contraceptive services.^20,21,22^ We have little evidence collected across the safety net system that also includes programs, such as Title X, that pay for contraceptive services in the safety net. Objective clinical data that capture the association of Medicaid expansion under the ACA with changes to the provision of contraceptive care across the safety net, regardless of insurance status or payer type, is lacking. Even small increases in the use of the most effective methods of contraception translate into a large outcome on unintended pregnancy, birth, and abortion rates,^23,24^ as well as public cost savings.

The purpose of this study is to examine the association of the ACA with quality of contraceptive care across the safety net system, using 2 newly developed metrics endorsed by the Office of Population Affairs (OPA) of the US Centers for Disease Control and Prevention and the National Quality Forum (NQF).^25,26^ These metrics^27^ are calculated annually at the individual level among women at risk of pregnancy in each measurement year: (1) receipt of a most effective or moderately effective contraceptive method and (2) receipt of a most effective contraceptive method. We use a patient-level data source that includes more than 300 CHCs across the US and assess overall rates of contraceptive provision, relative changes associated with Medicaid expansion (both immediate and longer term), and the role of Title X. We hypothesized that controlling for other programs and payers, the ACA would have a modest association with access to the most effective contraceptive methods. We compare outcomes in the preexpansion year to 2 points: one immediately postexpansion (2014) and a point longer after expansion (2016).

## Methods

We used electronic health record (EHR) data from a multistate network of CHCs and conducted a retrospective, cross-sectional, patient-level study. This analysis is part of the Reproductive Care in the Safety Net: Women’s Health after Affordable Care Act Implementation (EVERYWOMAN)^30^ study and estimates the association of ACA-sponsored Medicaid expansion with receipt of contraceptive care in the US safety net. This study follows the Strengthening the Reporting of Observational Studies in Epidemiology (STROBE) reporting guideline.

### Data Source and Sample

We used EHR data from the Accelerating Data Value Across a National Community Health Center Network (ADVANCE) clinical research network, a member of PCORnet, the National Patient-Centered Clinical Research Initiative.^28^ ADVANCE is a multicenter collaborative led by OCHIN Inc in partnership with Health Choice Network, Fenway Health, and Oregon Health & Science University. Outpatient EHR data from CHCs in the 3 data-sharing partner organizations (OCHIN Inc, Health Choice Network, and Fenway Health) are integrated and standardized into a common data model.^29^ ADVANCE data includes information from more than 5.6 million patients from CHCs across 30 states and is demographically similar to the national profile of patients in CHCs.^6^ ADVANCE data are collected under a waiver of authorization because of minimal risk to patients and the practical issues of getting consent from the number of patients included. More details on the data are in the previously published full study protocol.^30^ For this analysis, we included data from CHCs that were live (active) on their EHR and contributed data throughout the study period (2013, 2014, and 2016) and provided primary and preventive care services to women of reproductive age; these inclusion criteria resulted in CHC data from 20 states for analysis. The sample includes female patients aged 15 to 44 years old who were at risk of pregnancy.

### Dependent Variables: Receipt of Contraception

Our 2 primary outcomes are binary measures of receipt of contraception, based on the OPA and NQF contraceptive quality metrics specification^28^ and calculated annually at the individual level among women at risk of pregnancy in each measurement year: (1) receipt of a most effective or moderately effective contraceptive method (NQF 2903), and (2) receipt of a most effective contraceptive method (NQF 2904). The NQF standard 2903 includes oral contraceptive pills, injections, patches, rings, diaphragms, incident sterilization, or a long-acting reversible contraceptive (LARC) method (intrauterine device or implant). The NQF standard 2904 includes LARC methods (existing sterilization is an exclusion). These metrics are percentages of an eligible population calculated annually for women ages 15 to 44 years and reported in 2 strata: ages 15 to 20 years (adolescents) and ages 21 to 44 years (adults). We compared 2013 (before Medicaid expansion) with 2014 (immediately after Medicaid expansion) and 2016 (a longer time after Medicaid expansion).

**Figure 1.**
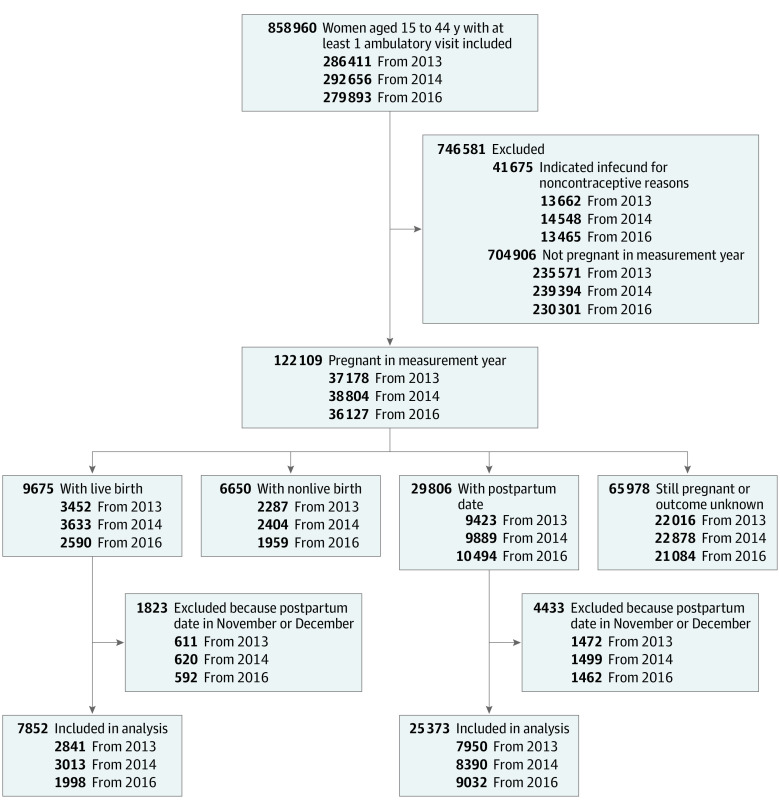
Study Population Selection

Women were eligible for a given measurement year if they were aged 15 to 44 years at the end of the year, had 1 or more ambulatory visit at an included study clinic during the year, and were at risk for pregnancy in the year. Women were excluded if they had evidence of previous sterilization noted in their EHR, were infecund for noncontraceptive reasons, or were pregnant in the measurement year and had a live birth delivery date in the last 2 months of the year (following OPA measure specifications^28^). Because the postpartum period is a key time for contraceptive care and delivery dates were sometimes unknown in the EHR, women were included in the sample if they were pregnant in the measurement year but had had a nonlive birth, had had a live birth in the first 10 months of the year, or had had an unknown pregnancy outcome. Delivery dates were missing in the outpatient EHR data for about 85% of pregnant women (11.5% of the study sample); we used postpartum visit dates as a proxy when available (accounting for about 25% of pregnant women). The remaining 60% of pregnant women with unknown pregnancy outcome were included in the final analysis because most would have delivered in the first 10 months of the year. We conducted a sensitivity analysis excluding all women with a pregnancy in the measurement year; results were unchanged (eTable 3 in the Supplement). Metric inclusion criteria are detailed in Figure 1.

### Independent Variable

Medicaid expansion status was our primary independent variable: our sample included patients from CHCs in 12 expansion states as of January 1, 2014 (California, Hawaii, Massachusetts, Maryland, Minnesota, New Mexico, Nevada, Ohio, Oregon, Rhode Island, Washington, and Wisconsin), and 8 nonexpansion states (Alaska, Florida, Indiana, Kansas, Missouri, Montana, North Carolina, and Texas). Wisconsin did not expand Medicaid eligibility to people with incomes of 138% FPL or less but opened adult enrollment up to those with incomes of 100% FPL or less, so it was categorized as an expansion state.^27,31^ Alaska, Indiana, and Montana implemented Medicaid expansion by January 1, 2016, and were reclassified as expansion states for the 2016 measurement year. Expansion vs nonexpansion status was based on the state location of each patient’s primary care facility.

We included patient-level, clinic-level, and state-level covariates in our patient-level analysis. We hypothesized that clinic-level and state-level factors could be associated with both Medicaid expansion and study outcomes.

### Patient-Level Covariates

We extracted patient-level covariates from the EHR, including age, race/ethnicity, and household income as a percentage of FPL. Hispanic ethnicity includes all patients with Spanish as a primary language, regardless of their coded ethnicities. Time-varying covariates (age and FPL) were assigned as of each patient’s last ambulatory visit in the given period. Where data elements were missing, we coded a separate missing/unknown category to not exclude these patients from our models.

Patients were assigned to a primary CHC clinic according to the most frequently visited facility in a given period. Individual-level insurance type was excluded from the analysis because Medicaid expansion status and having Medicaid are highly correlated and Medicaid coverage is in an associative pathway. Sensitivity analyses with insurance included as a covariate can be found in eTable 4 in the Supplement. To control for difference in health care utilization, we computed mean annual visit rates per for each measurement year among eligible patients and included an indicator for new patient status, because new patients are hypothesized to use care differently than patients with established care. We also controlled for binary pregnancy status (ie, pregnant or not pregnant in measurement year), because this affects both health care utilization and access to contraception.

### Clinic-Level Covariates

We identified patients who visited a Title X clinic^32^ within the measurement year because Title X clinics are known to provide broader range of contraceptive methods, including LARC.^22,33,34,35^ Title X funding status of the clinic was determined from data from the OPA.^36^ Urban vs rural status was assigned to each patient using Rural-Urban Commuting Area codes^37^ based on each patient’s primary clinic address. We also identified women who received care from a women’s health specialist.

### State-Level Covariates

We measured whether states used a Medicaid 1115 Waiver or State Plan Amendment to expand family planning coverage. In our data, these included expansion states California, Massachusetts, Maryland, Minnesota, New Mexico, Oregon, Rhode Island, Washington, and Wisconsin and nonexpansion states Florida, Indiana, Montana, and North Carolina.^38^ Because nearly all of the women had visits in states with a state family-planning program (approximately 90%; Table 1), we conducted a sensitivity analysis excluding state family-planning programs from our models. Results were unchanged. Based on previous literature and the potential confounding role on Medicaid expansion and contraceptive care, we still chose to include state family-planning programs in our final models.

**Table 1.** Study Population Characteristics by Medicaid Expansion Status

Characteristics	Medicaid, No. (%)		ASMD^a^
	Expansion states	Nonexpansion states	
Total eligible participants (before and/or after ACA), No.	310 132	235 408	
**Participant level**			
Age, y^b^			
Mean (SD)	28.9 (8.3)	29.1 (8.6)	.027
15-20	60 475 (19.5)	48 438 (20.5)	
21-25	58 475 (18.9)	40 973 (17.4)	.089
26-30	61 896 (19.9)	42 618 (18.1)	
31-35	52 059 (16.8)	42 618 (18.1)	
36-40	41 727 (13.5)	33 883 (14.4)	
41-44	35 501 (11.5)	30 069 (12.8)	
Race/ethnicity			
Hispanic	103 536 (33.4)	97 994 (41.6)	.581
Non-Hispanic			
White	144 110 (46.5)	63 455 (26.9)	
Black	33 026 (10.7)	63 877 (27.1)	
Other	18 826 (6.1)	5937 (2.5)	
Unknown	10 634 (3.4)	4145 (1.8)	
Income relative to federal poverty level			
≤138%	177 336 (57.2)	174 479 (74.1)	.477
>138%	36 741 (11.9)	32 203 (13.4)	
Unknown	96 055 (30.1)	28 726 (12.2)	
Insurance type^b^			
Medicaid	149 886 (48.3)	81 520 (34.6)	.432
Private	65 035 (20.1)	34 716 (14.6)	
Medicare or other public	15 828 (5.1)	31 933 (13.6)	
Uninsured	79 383 (25.6)	87 239 (37.1)	
New patient visit in study period^c^	100 948 (32.5)	96 362 (40.9)	.173
Pregnant in study period	42 668 (13.8)	38 243 (16.2)	.070
Title X visit in study period^d^	63 405 (20.4)	17 120 (7.3)	.387
Visit with women's health care professional	62 280 (20.1)	71 674 (30.4)	.240
**Clinic level**			
Urbanicity^d^			
Urbanized area	213 185 (68.7)	208 870 (88.7)	.497
Urban cluster	64 330 (20.7)	17 762 (7.6)	
Rural	32 617 (10.5)	8593 (3.6)	
Missing	0 (0.0)	183 (0.1)	
Annual No. of ambulatory visits			
Mean (SD)	3.6 (4.3)	3.0 (3.0)	.155
≤1	88 178 (28.4)	78 858 (33.5)	
>1 to 4	144 953 (46.7)	110 617 (47.0)	.145
>4 to 7	44 044 (14.2)	28 768 (12.2)	
>7	32 997 (10.6)	17 165 (7.3)	
**State level**			
State family planning program^d^	282 057 (90.9)	213 125 (90.5)	.014

Abbreviations: ACA, the Affordable Care Act; ASMD, average standardized mean difference.

^a^ All ASMD greater than 0.10 are significant (the values indicate marginal difference between distributions). All between-group *P* values were <.001 and are not reported.

^b^ Time-varying characteristics assigned as of last visit in study period.

^c^ Evaluation and management *Current Procedural Terminology* codes 99201 through 99205 and 99381 through 99387.

^d^ Based on each patient’s primary clinic.

### Statistical Analysis

We computed descriptive statistics for all eligible patients, stratified by expansion status and preexpansion and postexpansion periods. To assess whether patient samples differed substantially before vs after Medicaid expansion within the expansion group, we computed absolute standardized mean differences (ASMD). This effect size measure is used in observational studies to compare distributional differences between groups, with extensions to binary and multinomial variables.^39^ Importantly, ASMD is not affected by sample size (given our large sample size, most *P* values are highly significant) and can be used to compare distributions between overlapping (ie, nonindependent) groups as in this instance. To make comparisons between expansion status groups, we used ASMD to compare distributional differences in demographic and clinical measures. We considered an ASMD of more than 0.1 to denote marginal differences between the groups^40^ being compared.

To estimate association of the ACA with quality of contraceptive care, we used a difference-in-difference model.^41,42,43,44^ This quasiexperimental approach compares changes in contraceptive care outcomes at different points over time in states that expanded Medicaid vs those that did not. We calculated unadjusted and covariate adjusted estimates of the proportion of eligible women who received a most effective or moderately effective contraceptive method or a most effective method in preexpansion and postexpansion periods, by expansion status. We also stratified both outcomes by age groups (15-20 years and 21-44 years) and visiting vs not visiting a Title X clinic in the year. Adults made up most of our sample, and adult results were thus nearly identical to overall results; we therefore only show overall and adolescent results. We then fitted separate generalized estimating equation (GEE) models^45^ for each outcome and stratified by age groups with an identity link to obtain adjusted absolute percentage point differences comparing postexpansion vs preexpansion differences in contraceptive provision within expansion status groups, and difference-in-difference (DID)^46,47^ estimates with 95% CIs to test postexpansion vs preexpansion changes between expansion status groups. The GEE models included an indicator for measurement year (2013, 2014, and 2016), Medicaid expansion status, and the interaction between these variables (the difference-in-differences estimator). To help clarify comparisons, we used the SAS statement LSMEANS and contrast statements to output within-group before and after expansion and difference-in-differences estimates comparing each postexpansion year to the 2013 baseline year. Models were adjusted for covariates described above. The GEE models implemented a robust sandwich standard error estimator with a working independent correlation structure to account for the clustering of patients within CHCs. This study was approved by the Western Institutional Review Board. Analyses were conducted from May 2019 to February 2020 using SAS version 9.4 (SAS Institute). Statistical significance was set at *P *< .05 using 2-sided tests.

## Results

Our sample included 310 132 women from 315 CHCs in expansion states and 235 408 women from 165 CHCs in nonexpansion states. Compared with nonexpansion states, expansion states had a higher proportion of non-Hispanic white women (144 110 [46.5%] vs 63 455 [26.9%]; ASMD. 0.58), fewer patients with incomes greater than 138% of the FPL (36 741 [11.9%] vs 32 203 [13.4%]; ASMD, 0.48), and more patients with Medicaid (149 886 [48.3%] vs 81 520 [34.6%]; ASMD, 0.43; Table 1). Expansion states had more patients living in rural areas (32 617 [10.5%] vs 8593 [3.6%]; ASMD, 0.50) and more women with a Title X clinic visit (63 405 [20.4%] vs 17 120 [7.3%]; ASMD, 0.39). Within expansion groups, the populations who were eligible before and after ACA did not differ substantially, except on characteristics that would be expected to change (ie, ASMDs were >0.10 for age, insurance type, visit rates, and new patient status; Table 1 for all covariates and eTable 1 in the Supplement for full data before and after expansion by expansion status).

### Moderately Effective or Most Effective Contraception

Women in expansion states had an adjusted 24.4% receipt of moderately effective or most effective contraceptive methods in the year after Medicaid expansion (Table 2), an increase from 23.7% before expansion. Use of most effective or moderately effective contraceptives remained stable through 2016 (24.3%). Women in nonexpansion states experienced a greater adjusted increase in the receipt of moderately effective or most effective contraceptive methods in the immediate postexpansion period, from 17.9% to 19.6%. By 2016, receipt of contraceptives increased further, to 20.4%. The increase in coverage was thus significantly greater among women in nonexpansion states relative to expansion states (absolute adjusted difference-in-differences, 2014 vs 2013: −1.10 [95% CI, −1.88 to −0.33] percentage points; 2016 vs 2013: −1.93 [95% CI, −3.63 to −0.23] percentage points).

**Table 2.** Receipt of Contraception, Before Affordable Care Act Expansion (2013) vs Immediately (2014) and Longer (2016) After Expansion, by State-Level Medicaid Expansion Status

Characteristic	Expansion states			Nonexpansion states			Expansion vs nonexpansion status difference-in-differences estimate (95% CI)	
	2013	2014	2016	2013	2014	2016	2014 vs 2013	2016 vs 2013
**Full population, ages 15-44 y**								
No. of eligible women	161 720	164 467	162 656	108 945	111 522	101 718	NA	NA
Patients receiving moderately effective and most effective contraception^a^							NA	NA
No.	38 640	40 603	40 064	18 457	21 167	21 089	NA	NA
Adjusted %^b^	23.7	24.4	24.3	17.9	19.6	20.4	NA	NA
Adjusted % difference after vs before ACA^b^	0 [Reference]	0.64 (0.02-1.25)^c^	0.53 (−0.87 to 1.92)	0 [Reference]	1.74 (1.27-2.21)^d^	2.46 (1.52-3.41)^d^	−1.10 (−1.88 to −0.33)^c^	−1.93 (−3.63 to −0.23)^c^
Patients receiving most effective contraception^e^								
No.	6957	8706	9913	1805	2382	2774	NA	NA
Adjusted %^b^	4.4	5.3	6.1	1.8	2.2	2.4	NA	NA
Adjusted % difference after vs before ACA^b^	0 [Reference]	0.95 (0.61-1.29)^d^	1.76 (1.17-2.36)^d^	0 [Reference]	0.36 (0.04-0.68)^c^	0.58 (0.11-1.04)^c^	0.59 (0.13-1.05)^c^	1.19 (0.41-1.96)^c^
**Adolescents, ages 15-20 y**								
No. of eligible women	32 521	32 210	32 496	22 882	23 347	20 075	NA	NA
Patients receiving moderately effective and most effective contraception^a^								
No.	10 058	9872	9600	4056	4480	4410	NA	NA
Adjusted %^b^	28.9	28.4	27.8	21.2	22.4	24.1	NA	NA
Adjusted % difference after vs before ACA^b^	0 [Reference]	−0.45 (−1.39 to 0.48)	−1.11 (−2.60 to 0.37)	0 [Reference]	1.21 (0.39-2.02)^c^	2.85 (1.33-4.37)^d^	−1.66 (−2.89 to −0.42)^c^	−3.96 (−6.07 to −1.85)^d^
Patient receiving most effective contraception^e^								
No.	1381	1665	2042	290	328	395	NA	NA
Adjusted %^b^	4.0	4.9	6.1	1.80	1.87	2.10	NA	NA
Adjusted % difference after vs before ACA^b^	0 [Reference]	0.87 (0.49-1.25)^d^	2.09 (1.38-2.81)^d^	0 [Reference]	0.07 (−0.27 to 0.40)	0.30 (−0.15 to 0.76)	0.80 (0.30-1.30)^c^	1.79 (0.88-2.70)^d^

Abbreviations: ACA, the Affordable Care Act; NA, not applicable.

^a^ National Quality Forum standard 2903: receipt of oral contraceptive pills, injection, patch, ring, diaphragm, incident sterilization, or a long-acting reversible contraceptive method (intrauterine device or implant).

^b^ Preexpansion, postexpansion, and difference-in-difference estimates obtained from generalized estimating equation models clustered by primary clinic, assuming an independent correlation structure. Models adjusted for age, race/ethnicity, federal poverty level, urban or rural clinic location, visit rate, new patient status, Title X visit, care from a women's health care professional, presence of a state family-planning program, and pregnancy status. Data from after ACA were collected in 2014 or 2016; data before the ACA, 2013.

^c^ *P* < .05.

^d^ *P* < .01.

^e^ National Quality Forum standard 2904: receipt of long-acting reversible contraceptive methods (intrauterine device or implant).

### Most Effective Contraception (LARC)

After Medicaid expansion, annual use of most effective methods among women in expansion states increased from 4.4% in 2013 to 5.3% in 2014 and 6.1% in 2016 (Table 2). Women in nonexpansion states also experienced an increase in LARC in the postexpansion period, from 1.8% to 2.2% in 2014 and 2.4% in 2016. The increase in the most effective methods was thus greater among women in expansion states, particularly comparing 2016 with 2013 (absolute adjusted difference-in-difference, 1.19 [95% CI, 0.41-1.96] percentage points; Table 2).

### Adolescents

Among adolescents, use of moderately effective or most effective contraceptive methods did not change appreciably in expansion states (adjusted percentage receiving contraceptives in 2013, 28.9%; 2014, 28.4%; 2016, 27.8%; difference not significant). Adolescents in clinics in nonexpansion states experienced a small increase in use of moderately effective or most effective contraceptive methods in 2013, 2014, and 2016 (21.2%, 22.4%, and 24.1%, respectively). The increase in use of moderately effective or most effective methods was significantly larger among adolescents in nonexpansion states (2016 vs 2013: absolute difference-in-difference of −3.96 [95% CI, −6.07 to −1.85] percentage points; Table 2).

Adolescent use of most effective contraceptive methods increased nearly 1 percentage point in the first postexpansion year (from 4.0% to 4.9%) and more than 2 percentage points by 2016 (to 6.1%) in expansion states (Table 2). Adolescents seen in clinics in nonexpansion states experienced no significant change in the use of most effective methods (from 1.8% to 2.1% in each measurement year). The change in use of most effective methods was significantly greater among adolescents seen in clinics in expansion states in both the year immediately after ACA implementation (2014 vs 2013: absolute difference-in-difference, 0.80 [95% CI, 0.30-1.30] percentage points) and 3 years after ACA implementation (2016 vs 2013: absolute difference-in-difference, 1.79 [95% CI, 0.88-2.70] percentage points).

### Title X

**Figure 2.**
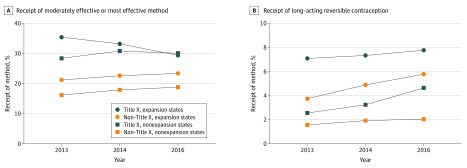
Adjusted Prevalence of Moderately Effective or Most Effective and Most Effective Effective Contraceptive Methods by Medicaid Expansion and Title X Status Standards for moderately effective or most effective contraceptive methods are as per National Quality Forum standard 2903, and standards for most effective contraceptive methods are as per National Quality Forum standard 2904.

Women who visited a Title X clinic in expansion states had higher percentages of receipt of both moderately effective or moderately effective and most effective contraceptive methods (Figure 2A). Compared with nonexpansion states, Medicaid expansion was significantly associated with an increase in the proportion of use of most effective methods use among women who were not seen in Title X clinics (Figure 2B), particularly 3 years after expansion (2016 vs 2013: adjusted difference-in-difference, 1.55 [95% CI 0.71-2.39] percentage points; eTable 2 in the Supplement).

## Discussion

We present new evidence that Medicaid expansion under the ACA is associated with an increase in use of the most effective contraception (LARC; intrauterine devices and implants) among women at risk of pregnancy, both immediately after expansion and at 3 years. This increase in effective contraceptive use attributable to Medicaid expansion was larger among a uniquely vulnerable population: adolescents seeking care in CHCs (who had a 2.1 percentage point increase in 2016 over 2013). While women in nonexpansion states experienced and increase in the access to moderately effective or most effective methods, they remained at overall lower levels of effective contraceptive use than women seen in expansion states. Although the absolute increase in contraceptive use attributable to Medicaid expansion is small, this difference is meaningful from a population health perspective. Being without insurance is a known risk factor for unintended pregnancy^48^; interventions that improve access to contraception for this population have profound population-level outcomes.^23,24^ Our findings persisted when accounting for other safety net programs, such as Title X. Medicaid expansion did not replace the benefits observed with Title X programs and can help improve access for women unable to visit a Title X clinic. We found that Medicaid expansion was associated with a 0.8 percentage point increase in access to LARC in 2014 relative to 2013, and this was 1.6 percentage points greater in 2016 vs 2013 among women seen in non–Title X clinics compared with those seen in nonexpansion states.

Unintended pregnancy disproportionately affects women with low incomes^5^; this is precisely the population included in our sample of CHCs that care for low-income and underserved populations. Previous work has reported conflicting results about provisions of the ACA, including the contraceptive mandate and Medicaid expansion, on contraceptive use.^49,50,51,52^ Our findings suggest that Medicaid expansion under the ACA improved access to the most effective contraception, LARC, among a population at high risk of unintended pregnancy. Furthermore, the observed increase in LARC in our sample of patients in safety net settings supports previous evidence that when cost barriers are removed, women choose LARC methods.^53^ Our findings that use of moderately effective methods decreased over time in expansion states is consistent with previous work using clinic-level reporting and survey data^18^ and may be explained by women switching to more effective methods. If this were the case, overall rates of use of moderately effective or most effective contraception would go down as most effective rates go up, given that most women in the group using moderately effective or most effective contraception are using moderately effective methods.

Our results show that, after Medicaid expansion, women in expansion states seeking care in CHCs had higher adjusted proportion of use of most effective methods than women with commercial insurance. Overall annual use of most effective methods was 2.3% (in nonexpansion states) or 5.3% (in expansion states), compared with 3.2% in a commercially insured population.^54^ Even small increases in LARC use, such as we observed in this study, translate into large changes in unintended pregnancy, birth, and abortion rates,^23,24^ as well as public cost saving in the US.

Adolescents who receive care in the safety net are at high risk for unintended pregnancy.^55^ In Medicaid expansion states, CHCs are providing adolescents better access to the most effective contraceptive methods compared with the commercially insured population. The adjusted proportion of provision of the most effective methods among adolescents was between 1.9% (in nonexpansion states) and 5.0% (in expansion states), compared with 2.4% among adolescents with commercial insurance in 2014.^54^

Our findings suggest that Medicaid expansion did not replace the influence of Title X in contraceptive care access but did increase access to most effective contraceptive methods for women who did not visit a Title X clinic. Research using clinic-level data has demonstrated that Title X clinics provide better access to LARC methods than non–Title X clinics.^22,56,57,58^ Historically, Title X has been the most important payer for contraceptive services for women with low incomes,^59^ and the exit of Planned Parenthood and individual states from the Title X program^34^ may intensify the role of non–Title X CHCs as publicly funded contraceptive care providers.^60^

Our results must be interpreted in the context of the goal of the OPA and NQF metrics; the intent of these metrics to track barriers to access to contraception. The moderately effective and most effective methods metric is useful to interpret in the context of published rates of use of these methods and estimates of the population at risk of unintended pregnancy. For example, we estimate that 24.3% of our adult sample received a moderately effective or most effective method in 2014. If we apply estimates of sexual activity and those actively seeking pregnancy from the National Survey of Family Growth,^61^ we calculate that 67.3% of contraceptive needs are met, leaving 32.7% opportunity for improvement. The LARC metric is intended to identify barriers to access, not to reach a threshold or promote use of LARC over other methods^61^; the National Survey of Family Growth comparisons are thus not used for this metric. We show that LARC use in the CHC population is similar to that of the commercially insured population but identify potential access gaps for women in this group who live in nonexpansion states compared with women with commercial insurance. Finally, this study focuses on 2 measures of access to contraceptive methods; while these are understood as quality metrics, the quality of contraceptive care is more than access to methods.^62^ New measures under review by the NQF assess the patient experience of care^63^ and will deepen our ability to assess the quality of contraceptive care across practice settings.

### Limitations

Our study has limitations. First, the CHC sample may not be generalizable to all patients in CHCs, CHC clinics, or states. However, our objective patient-level data come from the largest national data set on patients who receive care in safety net settings, and the ADVANCE patient population is demographically and clinically similar to the overall CHC population.^6^ Second, we had incomplete pregnancy outcome data for a subset of our sample; however, our sensitivity analyses found no changes to the main results. Similarly, missing data for covariates could result in bias, but the percentage of records with missing data was low. Third, we may not be able to fully identify women who had undergone sterilization prior to the study period; we looked back in the EHR with data available, but our observed sterilization rate (approximately 5%; Figure 1) is less than that reported in the National Survey of Family Growth.^61^ Fourth, the OPA and NQF metrics may not completely capture women at risk of unintended pregnancy (eg, they do not include a measure of sexual activity or pregnancy intention); however, the use of standard metrics to measure contraceptive access is an advance over previous studies. For quality metrics to be broadly used, they must be feasible to collect using existing administrative or clinical data.^64,65^ Finally, additional studies are needed to understand whether gains under Medicaid expansion were sustained past 2016.

## Conclusions

In conclusion, we found that ACA Medicaid expansion was associated with increased access to the most effective contraceptive methods across the safety net, especially for adolescents. Title X continues to play a vital role in contraceptive access for CHC populations.

## Notes

Supplement. **eTable 1.** Study population characteristics, pre- and post- expansion samples by Medicaid expansion status **eTable 2.** Receipt of contraception, pre-ACA (2013) vs immediate (2014) and longer-term (2016) post-ACA by Medicaid expansion and Title X status **eTable 3.** Sensitivity Analysis: Adjusted post- vs pre-ACA prevalence differences and difference-in-differences of contraception metrics among women without pregnancy in measurement year **eTable 4.** Receipt of contraception, pre-ACA (2013) vs immediate (2014) and longer-term (2016) post-ACA by Medicaid expansion status, including insurance covariate in models
