# Supplementary material for: Evaluation of Medicaid Expansion Under the Affordable Care Act and Contraceptive Care in US Community Health Centers
Source: JAMA Netw Open. 2020 Jun 4;3(6):e206874. doi: 10.1001/jamanetworkopen.2020.6874 (PMC7273194; doi:10.1001/jamanetworkopen.2020.6874)
Supplement: Supplement. — eTable 1. Study population characteristics, pre- and post- expansion samples by Medicaid expansion status eTable 2. Receipt of contraception, pre-ACA (2013) vs immediate (2014) and longer-term (2016) post-ACA by Medicaid expansion and Title X status eTable 3. Sensitivity Analysis: Adjusted post- vs pre-ACA prevalence differences and difference-in-differences of contraception metrics among women without pregnancy in measurement year eTable 4. Receipt of contraception, pre-ACA (2013) vs immediate (2014) and longer-term (2016) post-ACA by Medicaid expansion status, including insurance covariate in models [file jamanetwopen-3-e206874-s001.pdf]

Darney BG, Jacob RL, Hoopes M, et al. Evaluation of Medicaid expansion under the Affordable Care Act and contraceptive care in US community health centers. *JAMA Netw Open.* 2020;3(6):e206874. doi:10.1001/jamanetworkopen.2020.6874

eTable 1. Study population characteristics, pre- and post- expansion samples by Medicaid expansion status

eTable 2. Receipt of contraception, pre-ACA (2013) vs immediate (2014) and longer-term (2016) post-ACA by Medicaid expansion and Title X status

eTable 3. Sensitivity Analysis: Adjusted post- vs pre-ACA prevalence differences and difference-in-differences of contraception metrics among women without pregnancy in measurement year

eTable 4. Receipt of contraception, pre-ACA (2013) vs immediate (2014) and longer-term (2016) post-ACA by Medicaid expansion status, including insurance covariate in models

©2020 Darney BG et al. *JAMA Network Open*.

eTable 1. Study population characteristics, pre- and post- expansion samples by Medicaid expansion status

	**Medicaid expansion states** N (%)			**Medicaid non-expansion states** **N (%)**		
	Pre-ACA	Post-ACA	ASMD: within-group post vs pre	Pre-ACA	Post-ACA	ASMD: within-group post vs pre
Total eligible (pre- and/or post-ACA)	161,720	249,879		108,945	184,306	
Age[a]						
Mean (SD)	28.6 (8.2)	29.6 (8.3)	**0.124**	28.9 (8.5)	29.9 (8.6)	**0.120**
15-20	32,521 (20.1)	42,772 (17.1)	**0.118**	22,882 (21.0)	32,748 (17.8)	0.081
21-25	31,195 (19.3)	45,189 (18.1)		19,415 (17.8)	31,165 (16.9)	
26-30	32,290 (20.0)	50,664 (20.3)		19,450 (17.9)	34,076 (18.5)	
31-35	27,710 (17.1)	43,842 (17.6)		18,484 (17.0)	31,918 (17.3)	
36-40	22,152 (13.7)	35,800 (14.3)		15,939 (14.6)	27,922 (15.2)	
41-44	15,852 (9.8)	31,612 (12.7)		12,775 (11.7)	26,477 (14.4)	
Race/ethnicity			0.045			**0.115**
Hispanic	57,004 (35.3)	86,227 (34.5)		43,979 (40.4)	77,553 (42.1)	
Non-Hispanic white	74,555 (46.1)	113,692 (45.5)		29,072 (26.7)	48,896 (26.5)	
Non-Hispanic black	17,195 (10.6)	26,455 (10.6)		31,744 (29.1)	49,794 (27.0)	
Non-Hispanic other	8,870 (5.5)	15,260 (6.1)		2,571 (2.4)	4,826 (2.6)	
Unknown	4,096 (2.5)	8,245 (3.3)		1,579 (1.5)	3,237 (1.8)	
Federal poverty level			0.074			0.075
≤ 138%	96,712 (59.8)	143,235 (57.3)		83,456 (76.6)	136,617 (74.1)	
> 138%	15,789 (9.8)	30,911 (12.4)		13,715 (12.6)	25,543 (13.9)	
Unknown	49,219 (30.4)	75,733 (30.3)		11,774 (10.8)	22,146 (12.0)	
Insurance type[a]			**0.323**			**0.302**
Medicaid	65,214 (40.3)	129,289 (51.7)		36,587 (33.6)	66,287 (36.0)	
Private	29,356 (18.2)	53,440 (21.4)		8,977 (8.2)	30,626 (16.6)	
Other (Medicare, other public)	11,551 (7.1)	11,583 (4.6)		17,278 (15.9)	23,210 (12.6)	
Uninsured	55,599 (34.4)	55,567 (22.2)		46,103 (42.3)	64,183 (34.8)	
Urbanicity[b]			0.050			0.036

©2020 Darney BG et al. *JAMA Network Open*.

Urbanized area	109,354 (67.6)	172,510 (69.0)		94,991 (87.2)	163,336 (88.6)	
	**Medicaid expansion states** N (%)			**Medicaid non-expansion states N (%)**		
Urban cluster	35,348 (21.9)	50,890 (20.4)		9,368 (8.6)	14,102 (7.7)	
Rural	17,018 (10.5)	26,479 (10.6)		4,532 (4.2)	6,716 (3.6)	
Missing	0 (0.0)	0 (0.0)		54 (0.1)	152 (0.1)	
Annual number of ambulatory visits						
Mean (SD)	3.9 (5.0)	4.5 (4.2)	**0.127**	3.1 (3.4)	3.8 (3.0)	**0.212**
≤1	54,171 (33.5)	72,582 (30.4)	0.081	41,645 (38.2)	62,017 (34.9)	0.081
>1 - 4	66,793 (41.3)	104,933 (44.0)		46,241 (42.4)	79,874 (44.9)	
>4 - 7	21,371 (13.2)	32,215 (13.5)		12,379 (11.4)	20,982 (11.8)	
>7	19,385 (12.0)	28,729 (12.1)		8,680 (8.0)	15,095 (8.5)	
New patient visit in year[c]	41,842 (25.9)	81,158 (32.5)	**0.146**	37,905 (34.8)	75,471 (41.0)	**0.127**
Pregnant in year	33,647 (20.8)	52,238 (20.9)	0.060	8,431 (7.7)	14,270 (7.7)	0.096
Title X visit in year[b]	30,808 (19.1)	52,562 (21.0)	0.003	30,201 (27.7)	58,758 (31.9)	0
Visit with women's healthcare provider	20,080 (12.4)	36,147 (14.5)	0.050	15,014 (13.8)	31,758 (17.2)	0.091
State Family Planning Program[b]	148,013 (91.5)	227,435 (91.0)	0.018	97,910 (89.9)	166,302 (90.2)	0.012

Note. ASMD = Average Standardized Mean Difference; SD = Standard Deviation; **BOLD** = ASMD >0.10 (indicates marginal difference between distributions)

[a]Time-varying characteristics assigned as of last visit in study period

[b]Based on each patient's primary clinic

[c]Evaluation and management CPT codes 99201-99205, 99381-99387

©2020 Darney BG et al. *JAMA Network Open*.

eTable 2. Receipt of contraception, pre-ACA (2013) vs immediate (2014) and longer-term (2016) post-ACA by Medicaid expansion and Title X status

	Medicaid expansion states			Medicaid non-expansion states				
	**2013**	**2014**	**2016**	**2013**	**2014**	**2016**	**DID, 2014 vs 2013, expansion vs non-expansion (95% CI)**	**DID, 2016 vs 2013, expansion vs non-expansion (95% CI)**
**Title X clinics**								
N eligible women	33,647	34,401	33,943	8,431	9,235	9,400		
Moderately and most effective contraception[a]								
N received	11,834	11,338	10,095	2,475	2,773	2,842		
Adjusted[b] percentage of contraception receipt	35.2%	33.0%	29.7%	28.4%	30.8%	30.1%		
Adjusted[b] post- (2014 or 2016) vs pre-ACA (2013) percentage difference	Ref	-2.18 (-3.23, -1.14)**	-6.00 (-9.21, -2.80)**	Ref	2.41 (0.65, 4.17)*	1.75 (-0.66, 4.15)**	-4.59 (-6.69, -2.49)**	-7.75 (-11.91, -3.59)**
Most effective contraception[c]								
N received	2,315	2,443	2,627	287	333	492		
Adjusted[b] percentage of contraception receipt	7.1%	7.3%	7.8%	2.6%	3.2%	4.7%		
Adjusted[b] post- (2014 or 2016) vs pre-ACA (2013) percentage difference		0.24 (-0.44, 0.92)	0.68 (-0.53, 1.90)		0.68 (-0.41, 1.77)	2.09 (1.46, 2.72)**	-0.43 (-1.74, 0.87)	-1.40 (-2.77, 0.04)*
**Non-Title X clinics**								
N eligible women	128,073	130,066	128,713	100,514	102,287	92,318		
Moderately and most effective contraception[a]								
N received	26,806	29,265	29,969	15,982	18,394	18,247		
Adjusted[b] percentage of contraception receipt	21.2%	22.6%	23.4%	16.2%	17.9%	18.8%		
Adjusted[b] post- (2014 or 2016) vs pre-ACA (2013) percentage difference		1.41 (0.75, 2.07)**	2.22 (1.14, 3.31)**		1.71 (1.22, 2.19)**	2.57 (1.58, 3.56)**	-0.30 (-1.12, 0.53)	-0.34 (-1.18, 1.13)
	**Medicaid expansion states**			**Medicaid non-expansion states**				

©2020 Darney BG et al. *JAMA Network Open*.

	2013	2014	2016	2013	2014	2016	DID, 2014 vs 2013, expansion vs non-expansion (95% CI)	DID, 2016 vs 2013, expansion vs non-expansion (95% CI)
Most effective contraception[c]								
N received	4,642	6,263	7,286	1,518	2,049	2,282		
Adjusted[b] percentage of contraception receipt	3.8%	4.9%	5.8%	1.6%	1.9%	2.1%		
Adjusted[b] post- (2014 or 2016) vs pre-ACA (2013) percentage difference		1.15 (0.78, 1.51)**	2.03 (1.37, 2.69)**		0.36 (0.03, 0.69)*	0.48 (0.01, 0.95)*	0.79 (0.29, 1.28)*	1.55 (0.71, 2.39)**

NOTE. CI = confidence interval; DID = difference-in-difference; NQF = National Quality Forum
* p<.0.05
** p<.001

[a]NQF #2903: receipt of oral contraceptive pills, injection, patch, ring, diaphragm, incident sterilization, or a long-acting reversible contraceptive (LARC) method (intrauterine device or implant)
[b]Pre-post and difference-in-difference estimates obtained from GEE models clustered by primary clinic, assuming an independent correlation structure. Models adjusted for age, race, federal poverty level, urban/rural clinic, visit rate, new patient status, care from women's healthcare provider, presence of state family planning program, and pregnancy status
[c]NQF #2904: receipt of incident sterilization and LARC methods (intrauterine device or implant)

©2020 Darney BG et al. *JAMA Network Open*.

eTable 3. Sensitivity Analysis: Adjusted post- vs pre-ACA prevalence differences and difference-in-differences of contraception metrics among women without pregnancy in measurement year

	Medicaid expansion states		Medicaid non-expansion states			
	Adjusted absolute prevalence difference, 2014 vs 2013 (95% CI)	Adjusted absolute prevalence difference, 2016 vs 2013 (95% CI)	Adjusted absolute prevalence difference, 2014 vs 2013 (95% CI)	Adjusted absolute prevalence difference, 2016 vs 2013 (95% CI)	DID, 2014 vs 2013, expansion vs non-expansion (95% CI)	DID, 2016 vs 2013, expansion vs non-expansion (95% CI)
**Full population, ages 15-44**						
Moderately and most effective contraception[a]	0.65 (-0.01, 1.32)	0.63 (-0.83, 2.08)	1.93 (1.39, 2.47)**	2.78 (1.75, 3.81)**	-1.28 (-2.14, -0.42)*	-2.15 (-3.95, -0.36)*
Most effective contraception[b]	0.98 (0.61, 1.36)**	1.74 (1.21, 2.26)**	0.41 (0.13, 0.69)*	0.67 (0.23, 1.09)*	0.57 (0.10, 1.04)*	1.07 (0.38, 1.76)*
**Adolescents, ages 15-20**						
Moderately and most effective contraception[a]	-0.48 (-1.47, 0.52)	-0.96 (-2.49, 0.58)	1.30 (0.42, 2.17)*	3.02 (1.35, 4.68)**	-1.78 (-3.09, -0.46)*	-3.97 (-6.22, -1.72)**
Most effective contraception[b]	0.94 (0.56, 1.31)**	2.17 (1.49, 2.85)**	0.05 (-0.31, 0.41)	0.25 (-0.18, 0.68)	0.89 (0.37, 1.40)**	1.92 (1.06, 2.78)**
**Adults, ages 21-44**						
Moderately and most effective contraception[a]	1.06 (0.33, 1.80)*	1.10 (-0.54, 2.73)	2.19 (1.63, 2.74)**	2.56 (1.61, 3.51)**	-1.12 (-2.05, -0.20)*	-1.46 (-3.39, 0.47)
Most effective contraception[b]	1.00 (0.55, 1.46)**	1.63 (1.04, 2.21)**	0.53 (0.20, 0.85)*	0.76 (0.28, 1.25)*	0.48 (-0.09, 1.04)	0.87 (0.11, 1.63)*

NOTE. CI = confidence interval; DID = difference-in-difference; NQF = National Quality Forum

Difference and DID estimates obtained from GEE models clustered by primary clinic, assuming an independent correlation structure. Models adjusted for age, race, federal poverty level, urban/rural clinic, visit rate, new patient status, Title X visit, care from women's healthcare provider, and presence of state family planning program.

* p<.0.05

** p<.001

[a]NQF #2903: receipt of oral contraceptive pills, injection, patch, ring, diaphragm, incident sterilization, or a long-acting reversible contraceptive (LARC) method (intrauterine device or implant)

[b]NQF #2904: receipt of incident sterilization and LARC methods (intrauterine device or implant)

©2020 Darney BG et al. *JAMA Network Open*.

eTable 4. Receipt of contraception, pre-ACA (2013) vs immediate (2014) and longer-term (2016) post-ACA by Medicaid expansion status, including insurance covariate in models								
	**Medicaid expansion states**			**Medicaid non-expansion states**				
	**2013**	**2014**	**2016**	**2013**	**2014**	**2016**	**DID, 2014 vs 2013, expansion vs non-expansion (95% CI)**	**DID, 2016 vs 2013, expansion vs non-expansion (95% CI)**
**Full population, ages 15-44**								
N eligible women	161,720	164,467	162,656	108,945	111,522	101,718		
Moderately and most effective contraception[a]								
N received	38,640	40,603	40,064	18,457	21,167	21,089		
Adjusted[b] percentage of contraception receipt	23.9%	24.3%	24.1%	18.1%	19.7%	20.3%		
Adjusted[b] post- (2014 or 2016) vs pre-ACA (2013) percentage difference	Ref	0.42 (-0.14, 0.98)	0.14 (-0.12, 1.51)	Ref	1.54 (1.05, 2.03)**	2.14 (1.09, 3.19)**	-1.11 (-1.86, -0.38)*	-2.00 (-3.76, -0.25)*
Most effective contraception[c]								
N received	6,957	8,706	9,913	1,805	2,382	2,774		
Adjusted[b] percentage of contraception receipt	4.4%	5.3%	6.1%	1.9%	2.2%	2.4%		
Adjusted[b] post- (2014 or 2016) vs pre-ACA (2013) percentage difference		0.87 (0.16, 1.19)**	1.66 (1.09, 2.22)**		0.34 (0.01, 0.66)*	0.54 (0.03, 1.05)*	0.54 (0.10, 0.97)*	1.12 (0.35, 1.89)*

NOTE. CI = confidence interval; DID = difference-in-difference; NQF = National Quality Forum
* p<.0.05
** p<.001

[a]NQF #2903: receipt of oral contraceptive pills, injection, patch, ring, diaphragm, incident sterilization, or a long-acting reversible contraceptive (LARC) method (intrauterine device or implant)

[b]Pre-post and difference-in-difference estimates obtained from GEE models clustered by primary clinic, assuming an independent correlation structure. Models adjusted for age, race, federal poverty level, urban/rural clinic, visit rate, new patient status, care from women's healthcare provider, presence of state family planning program, pregnancy status, and insurance type.

[c]NQF #2904: receipt of incident sterilization and LARC methods (intrauterine device or implant)

©2020 Darney BG et al. *JAMA Network Open*.
